# Identification and Quantification of Gaps in Access to Autism Resources in the United States: An Infodemiological Study

**DOI:** 10.2196/13094

**Published:** 2019-07-10

**Authors:** Michael Ning, Jena Daniels, Jessey Schwartz, Kaitlyn Dunlap, Peter Washington, Haik Kalantarian, Michael Du, Dennis P Wall

**Affiliations:** 1 Division of Systems Medicine Department of Pediatrics Stanford University Stanford, CA United States; 2 Department of Biomedical Data Science Stanford University Stanford, CA United States

**Keywords:** autism, autism spectrum disorder, crowdsourcing, prevalence, resources, infodemiology, epidemiology

## Abstract

**Background:**

Autism affects 1 in every 59 children in the United States, according to estimates from the Centers for Disease Control and Prevention’s Autism and Developmental Disabilities Monitoring Network in 2018. Although similar rates of autism are reported in rural and urban areas, rural families report greater difficulty in accessing resources. An overwhelming number of families experience long waitlists for diagnostic and therapeutic services.

**Objective:**

The objective of this study was to accurately identify gaps in access to autism care using GapMap, a mobile platform that connects families with local resources while continuously collecting up-to-date autism resource epidemiological information.

**Methods:**

After being extracted from various databases, resources were deduplicated, validated, and allocated into 7 categories based on the keywords identified on the resource website. The average distance between the individuals from a simulated autism population and the nearest autism resource in our database was calculated for each US county. Resource load, an approximation of demand over supply for diagnostic resources, was calculated for each US county.

**Results:**

There are approximately 28,000 US resources validated on the GapMap database, each allocated into 1 or more of the 7 categories. States with the greatest distances to autism resources included Alaska, Nevada, Wyoming, Montana, and Arizona. Of the 7 resource categories, diagnostic resources were the most underrepresented, comprising only 8.83% (2472/28,003) of all resources. Alarmingly, 83.86% (2635/3142) of all US counties lacked any diagnostic resources. States with the highest diagnostic resource load included West Virginia, Kentucky, Maine, Mississippi, and New Mexico.

**Conclusions:**

Results from this study demonstrate the sparsity and uneven distribution of diagnostic resources in the United States, which may contribute to the lengthy waitlists and travel distances—barriers to be overcome to be able to receive diagnosis in specific regions. More data are needed on autism diagnosis demand to better quantify resource needs across the United States.

## Introduction

### Background

Autism spectrum disorder (ASD), a heterogeneous neurodevelopmental disorder characterized by repetitive and restricted behaviors and interests and social communication impairments, is the fastest growing developmental disorder in the United States [1]. The prevalence of ASD has increased by approximately 700% since 1990 and now affects 1 in 59 children [1-3].

Early access to diagnostic and therapeutic resources is essential for improving outcomes among children with ASD; however, families of individuals with autism often struggle to secure a diagnosis and find adequate therapeutic services [4-6]. The average age of diagnosis in the United States is estimated to be over 4 years [7], and about 27.1% of children remain undiagnosed at 8 years [8], with these figures being even higher among ethnic minorities [9]. Initial concerns are often already noted by families before children reach 2 years, making reliable diagnosis possible given adequate access to diagnostic resources [1,6,10-12]. Unfortunately, clinicians are often overburdened by the increasing population in need of care, creating waitlists exceeding 12 months for diagnosis. Waitlists are even longer for families in rural and underserved areas with a lower socioeconomic status, and an estimated 42% report having to travel to another city for a diagnosis [4,6,13,14]. A staggering 63% of families obtained a diagnosis for their child only after their third visit with a professional [6]. These delays in diagnosis and therapy leave many children untreated until after sensitive periods of development have passed [15-17]. Further complicating matters, treatment for ASD can cost a family up to US $100,000 annually, adding up to US $3.2 million in medical expenses over a lifetime [18,19]. More than half of all families report a lack of information and advice and little opportunity for parents to get involved in developing and reviewing treatment plans for their children [6,20].

### Objective

**Figure 1:**
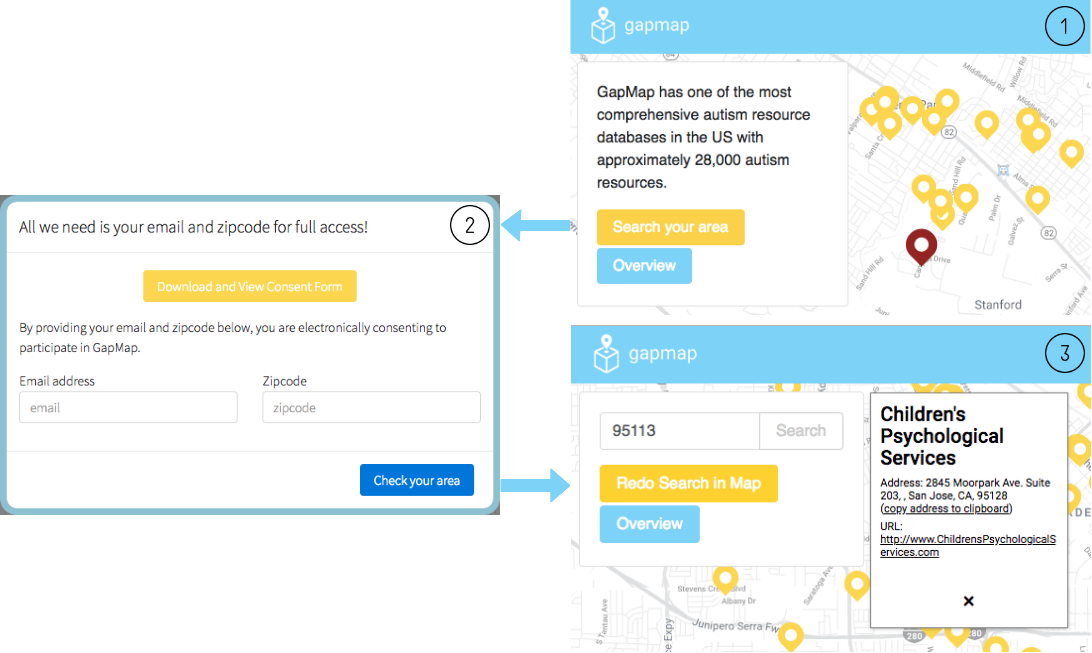
An example of the preliminary mapping interface for GapMap. (1) The landing page shows resources near Stanford University; (2) participants can electronically consent and participate from any desktop or mobile device; (3) after email and zip code submission, resources are shown near the zip code area.

Although there are indicators of significant resource shortages for families affected by autism in the United States [4-6], few studies have specifically explored autism resource epidemiology in the United States. Quantifying the expanding imbalance between clinicians and families in need of care and identifying the extent of gaps in access to autism resources are both essential steps toward providing support to individuals with autism. To address this need, we developed GapMap [21-23], a centralized crowd-powered Web platform that provides individuals affected by autism with colocated autism resources in their community (Figure 1).

Our preliminary analysis of data derived from GapMap has shown that in the United States, children who live closer to diagnostic centers are more likely to be diagnosed, highlighting a gap in access to care for families in rural communities [21]. This study expands upon these findings with additional data and analyses. In this study, we generated a comprehensive representation of autism resources in the United States, categorized each listed resource by service type, and examined more granular gaps in access in terms of distance and availability. For each of the 3142 counties in the United States, we estimated the average distance between individuals with autism and the nearest autism resource and approximated an annual demand over supply ratio of diagnostic resources.

## Methods

### GapMap

GapMap is connected to a MySQL database that stores autism resource metadata including, but not limited to, program names, addresses, service types, and geo-coordinates. GapMap’s front-end is written in React.js, and the back-end server runs on Amazon Web Services (AWS) application programming interface (API) Gateway, which connects to AWS Lambda and executes JavaScript packages to­­ communicate with our MySQL database hosted on Amazon Relational Database Service. GapMap allows users to search for resources near a given area and populates a map with markers representing each resource that expands into an information box containing its respective program name, address, and website. Due to its large set of resource geo-coordinates, the GapMap database provides the data needed to perform numerical analyses to further our understanding and characterization of resource epidemiology.

### Resource Database Validation and Categorization

The initial resource database for GapMap [21] was created in 2015 by mining 3 extensive and publicly available autism resource databases (eg, Autism Speaks [24], Autism Source [25], and Parents Helping Parents [26]). Each resource contained the following attributes: *Program Name*, *Program Description*, *Full Address*, *Phone*, *Email*, *URL*, *Latitude*, *Longitude*, and *Category*. Not all resources in GapMap’s original database were mappable, relevant, or up to date. To address this issue, a resource was discarded if it contained a post office box or an invalid physical address, was associated with a website that excluded keywords related to autism or was associated with a broken or missing website and lacked additional contact information. A resource with a broken or missing URL was unable to be validated; however, if an email address was provided, the resource was kept in the database for future validation and confirmation.

In an effort to increase comprehensiveness of the GapMap resource and to ensure it remains up to date, we used ParseHub [27], a third-party Web scraper, to extract an updated list of all resources listed on Autism Speaks. In addition, we extracted resources from the Google Places database using Google Places API [28] under the following search parameters: the keyword *autism*, city, state, and search radius. To ensure a comprehensive resource extraction, resources were searched within a 13-km radius of each city center. This radius includes the largest US city (Sitka, Alaska, which has a total area of 12,461 km^2^). The extracted resources from both databases were merged with the GapMap database, deduplicated based on geo-coordinates and program names, and processed through our validation pipeline.

We assigned resources in the GapMap database to 7 primary resource categories: *Diagnosis*, *Therapy*, *Health*, *Education*, *Recreation*, *Support*, and *Other*. These categories were determined by our clinical coordinators through comparisons between resource categories found on Autism Speaks, Autism Source, and Parents Helping Parents. Resources may include multiple resource categories under the *category* attribute. Table 1 maps the service type attributes already associated with a resource from its native database to 1 of our 7 primary resource categories. Websites associated with resources without a service type were scraped for keywords listed in Table 1 and allocated accordingly. Resources that were not captured by any of the keywords listed in Table 1, for entities such as "...Special Education Attorneys,” were allocated to the category *Other*.

**Table 1:** Keywords used to allocate resources to the Diagnosis, Therapy, Health, Education, Recreation, and Support categories on GapMap.

Keywords	Allocated category
Diagnosis, Where to get an Autism Diagnosis, Diagnostic, Assessments and Diagnosis	Diagnosis
Therapy; Early Intervention: Ages Birth-3; Interventions; Related Services; Early Intervention Services; Augmentative and Alternative Communication; Equine Therapy; Music Therapy; Occupational Therapy; Physical Therapy; Sensory Integration; Social Skills; Speech and Language Therapy; Applied Behavior Analysis (ABA); Floortime or DIR; Other Interventions; Picture Exchange Communication (PEC); Relationship Development Intervention (RDI); SCERTS Model; TEACCH; Verbal Behavior; Early Intervention; Related Services (Therapists); Speech/Language Therapy; Therapists; Art Therapy; instruction/intervention; Executive Function; ABA (Applied Behavior Analysis); Therapeutic; Speech	Therapy
Health, Biomedical Interventions; Health Services; Health and Dental Services; Diet or Nutrition; Other Biomedical Interventions; First Responder Resources; Dentists; Family Practitioners; Gastroenterologists; Inpatient Treatment Care Centers; Neurologists; Other Professionals; Pediatricians: Developmental, Pediatricians: General, Psychiatrists, Psychologists, State Mental Health Centers, Blood Draw or Phlebotomists; Community Mental Health Centers; Crisis Intervention Services; Substance Abuse Treatment; Crisis/Crime Victim Services; Dentist; Mental Health Professional; Other Medical Services; Physician; Doctors	Health
Education; Preschool Age: Ages 3-5; School Age: Ages 5-22; Post Secondary Education; Schools: Nonpublic (Private); Schools: Residential; Schools: Preschool; Transition to Adult Services; Academic Supports; Private/Non-Public School; Public School System; Job/Vocational; Transition; Living Skills; Schools; Tutoring; Academy; Preschool; Tutor; School; Learn; Teach; Pre-K	Education
Recreation; Recreational and Leisure Activities; Day Programs; Recreation and Community Activities; Camps; After-School Programs; Camps and Recreation; Social Activities; After School Care: Children; Recreation; Play	Recreation
Support; Support Groups; Community and Support Network; Advocates; Grandparents; Autism Speaks Communities; Local Autism Events; Local Autism Organizations; Military Family Resources; Online Support Groups; Religious Resources; Support Groups; Autism Society Affiliate (Chapter); Parent Training; Conferences; Other Local Organizations; Faith Community Services; Information and Support; Training; Support/Self Help Groups; Parent Support; Advocate-School District; Advocacy; Volunteer	Support

### Distance Between Resources and Individuals

As GapMap does not yet collect diagnostic information from users, we represented the population distribution of individuals with autism through a simulation. For each of the 3142 counties in the United States, geo-coordinates were generated in a random distribution inside a county bounding box derived from the 2010 US Census Bureau’s set of county Tiger/Line shapefiles [29]. The number of geo-coordinates representing the simulated population density was calculated by dividing the US Census Bureau’s 2016 County Population Estimate by 59 to normalize for the most recent autism prevalence rate [30]. This amounted to 5,476,742 coordinate points across the United States, where each of these points represents a simulated individual with autism.

All resources in the GapMap database were tagged with their respective geo-coordinates. The Euclidean distance was calculated between each geo-coordinate representing a simulated individual with autism and the geo-coordinate representing the autism resource from the GapMap database closest to the corresponding individual. These distances were used to calculate the average resource distance for each county. The values were then used to rank counties with gaps between autism resources and individuals with autism in descending order. This analysis was repeated for each of the 7 resource categories.

### Resource Load for Diagnostic Resources

Resource distance is correlated with the number of resources and the proportional distribution of resources in the region, but it is not an effective measure of how well resource demands are being met because of the variations in the number of individuals who can be served between resources. As a result, resource load was calculated. The diagnostic resources in the GapMap database comprises resources that provide medical diagnoses of ASD made by pediatricians, neurologists, and psychiatrists as well as diagnoses that occur through psychological assessments and mental health units. Resource load represents how well diagnostic centers can meet the demands of the individuals in a given county annually. Ideally, this value would be a demand over supply ratio of diagnostic services in a given county annually; however, because of the lack of available data on individuals diagnosed and waitlisted for diagnosis for each state, an approximation of this measure was represented by a resource load formula. In addition, as calculating resource load for each diagnostic center rather than region would lead to overestimated values from the potential recounting of individuals within the proximity of multiple diagnostic resources, the previous resource load formula was adjusted to calculate a comparative value that approximates the demand over supply ratio of autism resources. The new resource load below does not exhibit any meaning as a stand-alone value but can be compared with other state resource load values to estimate how well diagnostic resources can meet individual demand.

The resource load *RL_c*, as computed for diagnostic resources for each of the 3142 US counties, is represented by this formula:

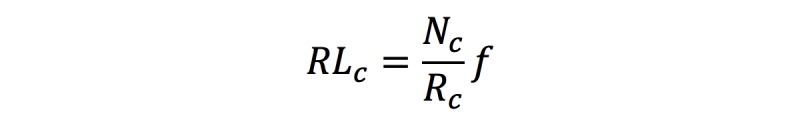



*N_c*, the county population, is derived from the US Census Bureau’s 2016 County Population Estimate [30]. *R_c* is the number of diagnostic resources in a given county from the GapMap database. For a more intuitive comparison of resource load values, *f* is the ratio selected to normalize by the lowest state resource load, resulting in a value of 1 for the lowest state resource load. A higher resource load value may suggest that resources in a given region are less likely to meet demand.

## Results

### Resource Database Validation and Categorization

We originally identified 29,935 autism resources in the United States and removed 8402 resources from the GapMap database through our validation and deduplication process. Additional resources were then extracted from Autism Speaks and Google Places, verified through our validation pipeline, merged with our database, and deduplicated, resulting in the addition of 6470 resources and amounting to a total of 28,003 unique and validated resources in the GapMap database. Table 2 shows the allocation of all resources into each of the 7 resource categories. Resources may be allocated to multiple categories.

### Distance Between Resources and Individuals

**Figure 2:**
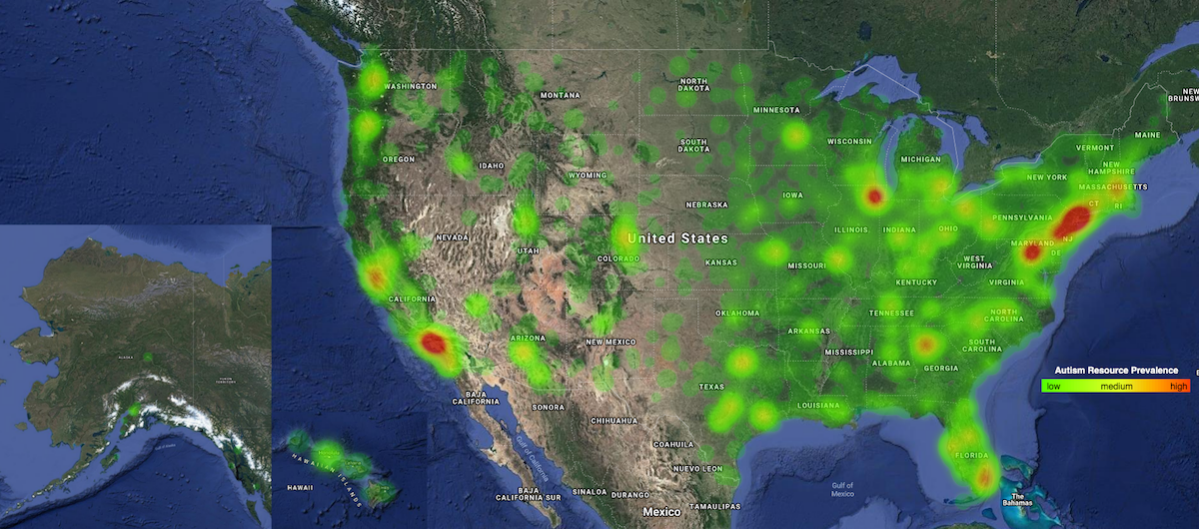
A heat map depicting the density and distribution of autism resources across the United States (Alaska and Hawaii not to scale), where red indicates a high density of resources.

The estimated average distance between an individual with autism and the nearest autism resource belonging to any of the 7 resource categories across the United States is 17.12 km. Table 2 outlines the average distance of the nearest resource specific to each category. Due to the large number of US counties, Table 3 only shows the 10 states with the largest and smallest average distances to the nearest resource, respectively. On average across the United States, individuals are over 21 km away from resources that belong to the *Therapy* category and are farthest away from resources that belong to the *Diagnosis* category, at over 35 km. Figure 2 represents a heat map of the overall density of resources across the United States. As shown in the map, resources are sparsely distributed across the western half of the country and are densely crowded surrounding regions of higher population density.

**Table 2:** The number, percent breakdown, and average distance of autism resources for each of the 7 resource categories.

Category	Resources, n (%)	Average distance (km)
All resources	28,003 (100.00)	17.12
Therapy	11,602 (41.43)	21.84
Support	8153 (29.11)	21.65
Health	7236 (25.84)	24.02
Education	5595 (19.98)	24.17
Recreation	2791 (9.97)	30.44
Diagnosis	2472 (8.83)	35.49
Other	5190 (18.53)	26.80

**Table 3:** Top 10 states in the United States with the largest and smallest average distances between an individual with autism and the nearest autism resource, respectively.

Top 10 states with largest distance		Top 10 states with smallest distance	
State	Distance (km)	State	Distance (km)
Alaska	100.66	New Jersey	3.74
Nevada	54.16	Connecticut	4.67
Wyoming	54.14	Maryland	5.65
Montana	49.12	Massachusetts	6.42
Arizona	43.69	New York	7.07
North Dakota	41.52	Rhode Island	7.20
New Mexico	36.75	Pennsylvania	7.60
South Dakota	33.24	Delaware	8.09
Oregon	27.96	Virginia	9.06
Idaho	27.35	New Hampshire	9.3

### Resource Load for Diagnostic Resources

Of the 3142 US counties surveyed, our analysis found that 2635 counties (83.86%) did not have a single diagnostic resource. Table 4 shows the average resource load for the 10 states in the United States with the highest and lowest resource loads, respectively.

**Table 4:** Top 10 states in the United States with the highest and lowest resource load, respectively.

Top 10 states with the highest load		Top 10 states with the lowest load	
State	Resource load	State	Resource load
West Virginia	5.71	Montana	1
Kentucky	4.26	Connecticut	1.09
Maine	4.15	Colorado	1.10
Mississippi	3.73	Rhode Island	1.10
New Mexico	3.71	New Jersey	1.14
Oklahoma	3.49	Pennsylvania	1.18
South Carolina	3.26	Massachusetts	1.25
Nevada	2.82	Wisconsin	1.26
Tennessee	2.68	Maryland	1.32
Nebraska	2.64	New York	1.32

## Discussion

### Principal Findings

In this study, we utilized GapMap to measure autism resource accessibility and availability across the United States. GapMap’s database contains 28,003 unique, validated, and categorized autism resources. As shown in Table 2, average distance is negatively correlated with the number of resources, with the exception of *Therapy* and *Support* resources. This suggests that *Support* resources are more evenly distributed around the population than *Therapy* resources. These data show an uneven distribution between resources, which calls for the need to replace simulated population data of families affected by autism with actual population data. This will help service providers strategically allocate resources in locations, which would allow a distribution of services commensurate with the population in need.

The average distance between the nearest resource belonging to any resource category and a family affected by autism is 17.12 km. As resources that belong to an individual resource category are a subset of the total number of resources, the average distances corresponding to each individual category will be greater than 17.12 km, as the average distance is negatively correlated with the number of resources. The *Therapy* resource category contained the most resources (41.43% [11,602/28,003] of all resources on GapMap) and was tied with the *Support* resource category for the lowest average distance to families, at 21 km. The *Diagnosis* resource category contained the fewest resources (8.83% [2472/28,003] of all resources in GapMap) and had the greatest average distance (35.49 km) to individuals with autism, providing some support for the diagnostic bottleneck often reported by parents [12,14,31]. This bottleneck may be largely attributed to the lack of qualified specialists who can perform an autism diagnosis. It is estimated that there are only 8300 child psychiatrists, 1500 child neurologists, and 1000 developmental-behavioral pediatricians in the United States [32]. Specialists working with autism are even scarcer, in part, because of the lack of monetary incentives compared with other specialties; developmental-behavioral pediatricians undergo extra years of training through fellowship programs but earn equal or less than primary care physicians [14].

Of the 3142 US counties, only 507 have 1 or more diagnostic resources, suggesting that centers are not only being overloaded by its county population but also by individuals outside of the county with no access to diagnostic resources locally. This observation aligns with the historical lack of mental and behavioral health resources in rural communities [33,34]. In contrast, a state such as California no longer requires an autism diagnosis to access behavioral health treatment: families and individuals with autism may seek therapy services while waiting for a diagnosis. Further research via GapMap presents an opportunity to enhance knowledge surrounding resource use, autism prevalence, and resource availability specifically in rural areas.

In a study of several countries including the United States, Williams et al found urban locations reported higher prevalence estimates than those reported in rural and mixed locations [35], and research in Denmark showed a higher likelihood of early diagnosis and treatment in urban settings [36]. These data suggest that autism is underdiagnosed and therefore possibly treated later in rural settings. Unlike traditional epidemiological studies, GapMap is also able to accurately measure autism prevalence and resource availability across the United States in rural and urban locations, as families and service providers can directly access this database from their homes and provide the information needed. Furthermore, GapMap provides a robust, user-friendly autism resource database that depicts the real-time availability of resources for individuals with autism across the country.

### Limitations

Although we made significant programmatic and manual effort to ensure the accuracy and completeness of the autism resources contained in GapMap, the true number of autism resources in the United States may be above or below the estimated value we derived through our analysis. As we do not have a method to ensure homogeneous reporting of resources by the primary databases from which we extracted our resources, the GapMap database is prone to population biases as a result of increased probability of reporting in densely crowded areas. Due to the lack of complete locational data on the population affected by autism in the United States, we assumed a randomly distributed simulation of the autism population. The geographic boundaries used to simulate the population are rectangular bounding boxes, which do not perfectly capture the actual geographic boundaries that exist in the US counties; however, we found that this primarily affects the relatively small number of counties near bodies of water. Furthermore, the resource distances are calculated with Euclidean distance, which may underestimate the true resource distance. Finally, because of a lack of annual data on the number of individuals who received an autism diagnosis and who were waitlisted for diagnosis for each state, we used a comparative resource load value, allowing only for comparison within this analysis.

### Conclusions and Future Directions

These results show an uneven distribution of autism resources throughout the United States and that diagnostic resources are the most underrepresented out of all autism resource categories analyzed. Demand for diagnostic resources varies throughout the United States, contributing to the diagnostic bottleneck, long waitlists spanning several months, and large travel distances for families in rural or underserved locations. The discrepancy in access to services across the United States could be attributed to many factors. We will attempt to parse such sources further as more granular population data are collected from families affected by autism.

**Figure 3:**
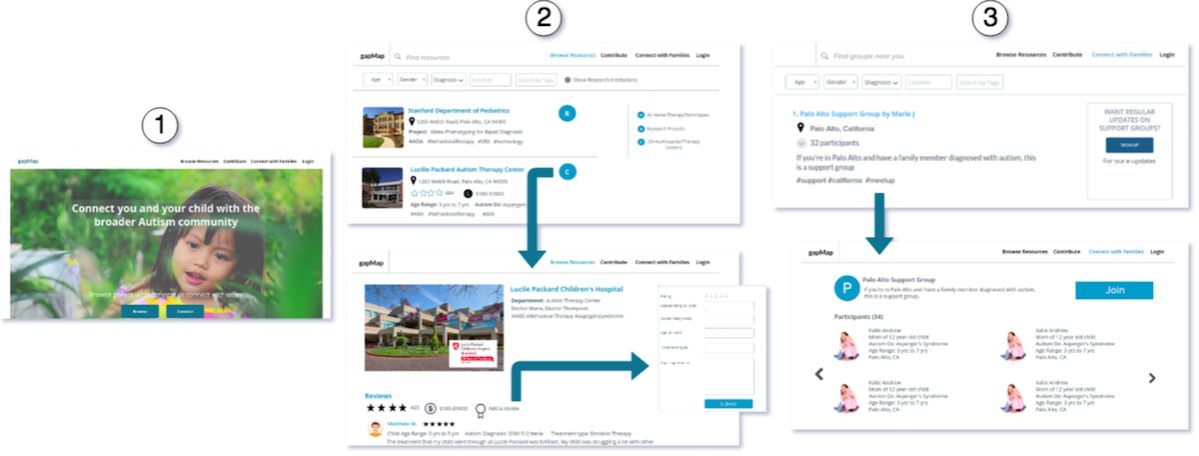
Depiction of the proposed rating- and review-based features. (1) Landing page; (2) allows families to rate and review resources; (3) allows families to join local autism communities created by other families.

Consequently, GapMap will now endeavor to collect geocoded location data and self-reported diagnoses from recruited families. Over time, this will enable a more detailed and accurate representation of gaps in access to care by enabling us to replace simulated family locations with self-reported locations. Euclidean distances between individuals and resources will be replaced with more accurate road distances that factor in street network using Google Maps API. In addition, we plan to incorporate several new features to the GapMap platform (Figure 3), which will allow families to interact with each other by rating resources, leaving reviews, adding additional resources, and submitting wait times for services. This will serve as a system that evaluates resources in terms of quality, thus providing greater incentive for families to join, while collecting real population data needed to improve autism resource epidemiological research.

## Abbreviations

API: application programming interface
ASD: Autism Spectrum Disorder
AWS: Amazon Web Services
